# Early Detection of Bronchial Lesions Using System of Autofluorescence Endoscopy (SAFE) 1000

**DOI:** 10.1155/DTE.5.99

**Published:** 1999

**Authors:** Masatoshi Kakihana, Kim Kyong Il., Tetsuya Okunaka, Kinya Furukawa, Takashi Hirano, Chimori Konaka, Harubumi kato, Yoshiro Ebihara

**Affiliations:** 1 Department of Surgery Tokyo Medical University 6-7-1 Nishishinjuku Shinjuku-ku Tokyo 160-0023 Japan; 2 Department of Pathology Tokyo Medical University 6-7-1 Nishishinjuku Shinjuku-ku Tokyo 160-0023 Japan

## Abstract

Recently several endoscopic fluorescence detection systems have been developed. In some of them, laser light was used for the excitation of autofluorescence, and sophisticated techniques were also necessary to amplify the fluorescence signal as well.

The result of fluorescence diagnosis using a simple system with a conventional Xenon lamp excitation and an image intensifier is reported. The respective results of sensitivity and positive predictive values of cancer plus dysplasia were 66%, and 62% by standard bronchoscopy and 92% and 88% by the newly developed autofluorescence system. In this paper, developed endoscope for detection of tissue/mucosal autofluorescence without the application of any photosensitizing agents or use of any lasers is evaluated.


agnostic and Therapeutic Endoscopy, Vol. 5, pp. 99–104
:prints available directly from the publisher
otocopying permitted by license only

© 1999 OPA (Overseas Publishers Association) N.V.
Published by license under
the Harwood Academic Publishers imprint,
part of The Gordon and Breach Publishing Group.
Printed in Singapore.

### Early Detection of Bronchial Lesions Using System of Autofluorescence Endoscopy (SAFE) 1000

MASATOSHI KAKIHANA[a,*], KIM KYONG IL.[a], TETSUYA OKUNAKA[a], KINYA FURUKAWA[a], TAKASHI HIRANO[a], CHIMORI KONAKA[a], HARUBUMI KATO[a] and YOSHIRO EBIHARA[b]

[a]*Department of Surgery*, [b]*Department of Pathology, Tokyo Medical University, 6-7-1 Nishishinjuku, Shinjuku-ku, Tokyo, 160-0023, Japan*

**Recently several endoscopic fluorescence detection systems have been developed. In some of them, laser light was used for the excitation of autofluorescence, and sophisticated techniques were also necessary to amplify the fluorescence signal as well.**

**The result of fluorescence diagnosis using a simple system with a conventional Xenon lamp excitation and an image intensifier is reported. The respective results of sensitivity and positive predictive values of cancer plus dysplasia were 66%, and 62% by standard bronchoscopy and 92% and 88% by the newly developed autofluorescence system. In this paper, developed endoscope for detection of tissue/mucosal autofluorescence without the application of any photosensitizing agents or use of any lasers is evaluated.**

*Keywords:* Autofluorescence diagnosis, Dysplasia, Early cancer

### INTRODUCTION

The number of early stage lung cancer has increased in recent years in Japan, mainly due to the common use of sputum cytology for high risk patients or symptomatic patients in Japan. If central type lung cancer is detected at an early stage, endoscopic treatment, especially photodynamic therapy can obtain a favorable therapeutic response [1–3], thus early detection is a goal of leading facilities in the world. Since central type early stage lung cancer shows no abnormality on chest X-ray or even on helical CT, endoscopy is the only method to detect these lesions. However, early cancer, especially carcinoma *in situ*, shows only subtle mucosal changes [4], thus repeated examinations are sometimes necessary to localize the lesion [5]. When localization fails in cases with positive sputum cytology, blind brushing or washing cytology examination of segmental or subsegmental bronchi is commonly performed [6]. Photodynamic diagnosis is another approach to detect these lesions [7] but photosensitizer cannot be used routinely. To overcome this problem, Lam and Palcic created a fluorescence detection system [8–11]. The principle of this system is based on the phenomenon that the intensity of autofluorescence of tumor is different from that of normal tissue illuminated by light with

* Corresponding author. Tel.: +81 3 3342 6111 (Ext. 5070). Fax: +81 3 3349 0326.

a specific wavelength. Their Lung Imaging Fluorescence Endoscope (LIFE) system employs laser light for the excitation of autofluorescence, and sophisticated techniques to amplify the fluorescence sign [10,11]. Even experienced bronchoscopists sometimes fail to localize dysplastic lesions, in which bronchial mucosal changes are extremely subtle. In the multistep carcinogenesis theory, dysplasia has been considered as one of the precancerous status, which we have proved by animal experiments and immunohistochemical studies [12,13].

The objective of the present study is to judge if a simple fluorescence endoscopy system with a conventional Xenon lamp could be useful in detecting early cancer and dysplasia of the bronchus.

### MATERIALS AND METHODS

### Fluorescence Endoscope

A prototype of the System of Autofluorescence Endoscopy (SAFE-1000, Asahi Optical Co., Ltd., Tokyo, Japan) was used in this study. A conventional Xenon light equipped with a special filter was used as an excitation light source instead of laser light. Infrared light was eliminated by the infrared-cut filter and only 420–480 nm excitation light was delivered through an excitation filter and transmitted via an image guide. The intensified autofluorescence of normal mucosa appeared green on the image monitor, although abnormal areas showed a cold image caused by the lack of autofluorescence. The camera which contained a fluorescence filter, image intensifier, and a TV camera was attached to the eyepiece of the bronchoscope. Only 520–600 nm light passed through the fluorescence filter, and this autofluorescence was amplified by the image intensifier.

The intensified image is processed simultaneously on the video monitor in real time [14].

### Subjects

A total of 72 cases were studied and 147 sites were biopsied from January 1997 to June 1997 in Tokyo Medical University Hospital. The subjects were classified into three categories; cases with lung cancer, 42 cases (58%), abnormal sputum cytology findings, 16 cases (22%) and smokers with recurrent symptoms, 14 cases (19%), respectively. Informed consent was obtained in all cases. At first, conventional bronchoscopy (C.B.) using an FB18RX (Asahi Optical Co., Ltd., Tokyo, Japan) was performed under local anesthesia and areas suspicious for cancer or dysplasia were noted for subsequent biopsy. After the C.B. procedure, the bronchoscope was placed in the trachea of the patient, fluorescence examination was begun by attaching a fluorescence camera to the eye-piece of the bronchoscope and by attaching the infrared filter to the Xenon lamp. Areas with decreased green autofluorescence were objectively noticed and diagnosed as suspicious by bronchoscopists. The biopsy were taken from all abnormal areas discovered by the C.B. or SAFE, or both. In this study, negative control biopsies (C.B. normal and SAFE normal) were not routinely obtained. Biopsy Specimens were interpreted by an experienced pathologist and pathological diagnosis was the gold standard to decide the diagnostic accuracy.

### RESULTS

A total of 72 subjects were enrolled in this study; 48 men and 24 women, aged 49–79 (Table I). The enrolled patients were classified into three groups. The first group consisted of 42 patients (58%) with known lung cancer. In 24 patients, primary lesions were visible by both C.B. and SAFE, but in the other 18 cases, tumors were not recognized because the primary lesions were located in periphery of the lung. The results of spectral analysis with SAFE of an invasive squamous cell carcinoma at the orifice of the right middle lobe bronchus is shown in Fig. 1. The difference of fluorescence intensity between normal and tumor tissue was detected at 500–600 nm wavelength.

The second group consisted of 16 cases (22%) with abnormal sputum cytology findings but normal chest X-ray. Early cancer was detected in 4 cases, dysplasia was diagnosed in 9 cases but the lesion was unknown in 3 cases.

The third group consisted of 14 cases (19%), all of whom currently smoked, with a history of more than 20 pack-years. Dysplasia was found in 2 cases, chronic inflammation in 10 cases, and the rest of 2 patients were pathologically normal.

TABLE I Characteristics of enrolled patients (72 cases)

| | |
|---|---|
| I. Lung cancer (42 cases) | |
| Endoscopically recognized/unrecognized | 24/18 |
| Squamous cell carcinoma | 20 |
| Adenocarcinoma | 21 |
| Small cell carcinoma | 1 |
| Dysplasia was found in 11 cases (26%) | |
| II. Abnormal sputum cytology findings (16 cases) | |
| Early cancer | 4 |
| Dysplasia | 9 |
| Origin unknown | 3 |
| III. Smokers with symptoms (14 cases) | |
| Dysplasia | 2 |
| Normal/inflammation | 12 |

A total of 147 sites were biopsied. A comparison of endoscopic findings and pathological diagnosis of the biopsy specimens is shown in Table II. Invasive cancer was detected at 24 sites (16%), all of which were correctly diagnosed by both C.B. and SAFE. Early cancer was detected at 4 sites (6%), 3 of them were recognized by both C.B. and SAFE. The remaining site was almost unrecognized by C.B. but objectively recognized by SAFE. Figure 2 shows the endoscopic findings of this case, a 70 year-old man, who had been referred to our clinic because of abnormal sputum cytology findings. A cold spot was recognized at the orifice of left B6 by SAFE, while C.B. findings were almost normal. Biopsy revealed squamous cell carcinoma.

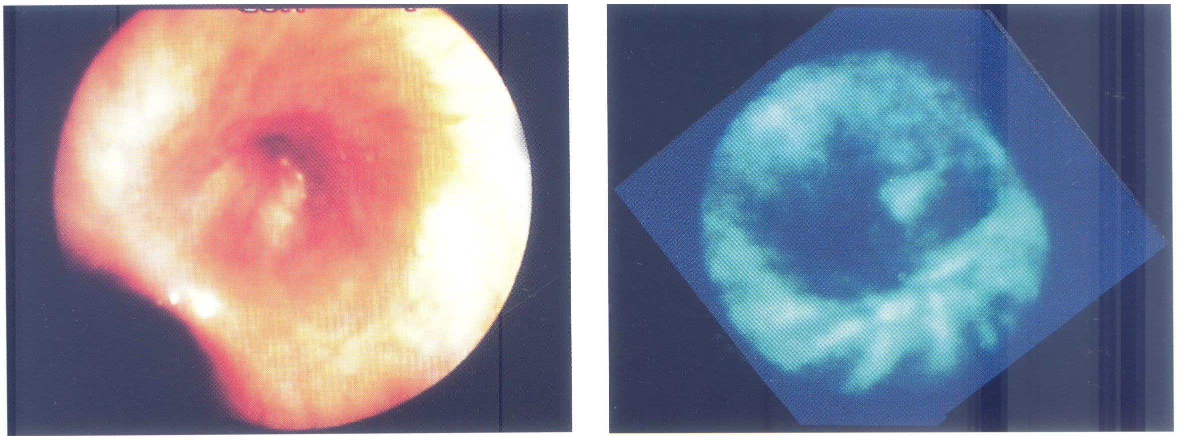

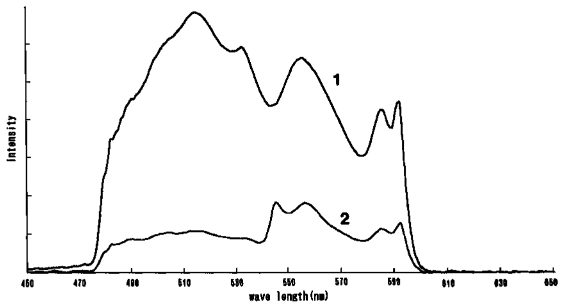

FIGURE 1 Spectrum analysis of normal and tumor tissue was performed during fluorescence examination of cases with invasive squamous cell cancer. The intensity of autofluorescence decreased in tumor tissue compared with that of normal tissue excited at 440 nm. (1: normal, 2: tumor).

TABLE II Results of endoscopic findings and pathology (147 sites)

| | SAFE negative | SAFE positive |
|---|---|---|
| Invasive cancer ($n=24$) | | |
| CB positive | | 24 (100%) |
| Early cancer ($n=4$) | | |
| CB negative | | 1 (25%) |
| CB positive | | 3 (75%) |
| Dysplasia ($n=51$) | | |
| CB negative | 0 (0%) | 25 (49%) |
| CB positive | 6 (12%) | 20 (39%) |
| Normal/Inflammation ($n=68$) | | |
| CB negative | 22 (32%) | 15 (22%) |
| CB positive | 16 (24%) | 15 (22%) |

A total of 51 sites were pathologically diagnosed for dysplasia. C.B. diagnosed 26 sites (specificity: 51%) and SAFE diagnosed 45 sites (specificity: 88%). Figure 3 shows a typical case of dysplasia negative on C.B. negative, and positive on SAFE.

This case, a 67 year-old man, was referred to our hospital for detailed examination of abnormal sputum cytology findings. The cold spot was noted in right B5 by SAFE, although C.B. findings were normal. Biopsy revealed moderatly differentiated dysplasia. In relation to sensitivity, C.B. showed

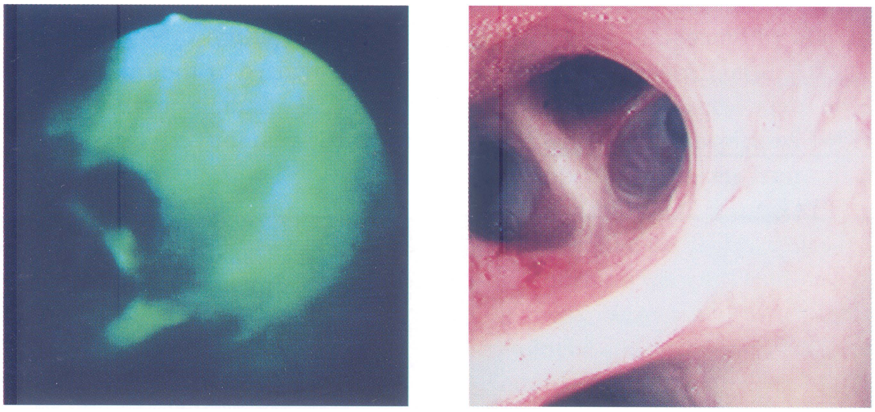

FIGURE 2 Conventional and fluorescence bronchoscopic findings of early squamous cell carcinoma in left B6. The lesion was recognized by SAFE although C.B. findings were normal.

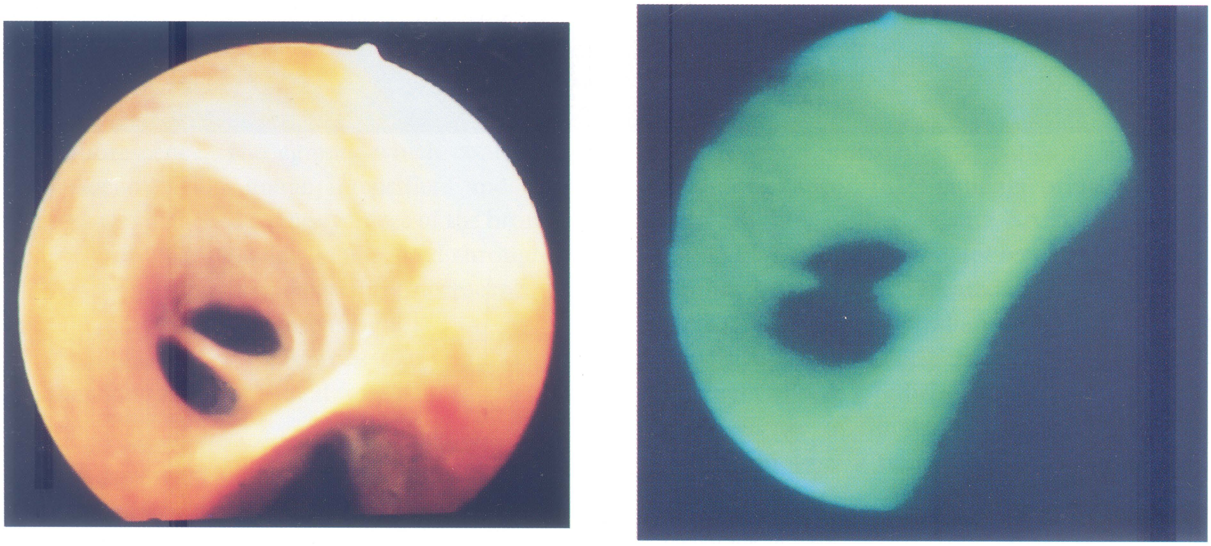

FIGURE 3 Conventional and fluorescence bronchoscopic findings of dysplasia in right B5. The lesion was recognized by the SAFE although C.B. findings were normal.

TABLE III Summary of clinical data

| | CB (%) | SAFE (%) |
|---|---|---|
| Sensitivity | | |
| Cancer and dysplasia | 66 | 92 |
| Dysplasia | 51 | 88 |
| Positive predictive value | 62 | 71 |
| Specificity | 54 | 56 |

54% and the SAFE system, 56%. The summary of diagnostic rate is shown in Table III.

### DISCUSSION

Recent advances in molecular biology suggest a multistep theory of carcinogenesis [15]. Thiberville reported genetic losses in progressive precancerous lesions. Also Hirano *et al.* reported hyperproliferation high frequency p53 abnormality in dysplasia and early squamous cell carcinoma [13]. As dysplasia might be one premalignant condition, early detection and follow up of these lesions are essential. Chemoprevention with vitamin B12 was effective in reversing persistent dysplasia to normal status [16]. Photodynamic therapy was successful in the treatment of central type early stage lung cancer and it was approved to be covered by the National Health Insurance in Japan [17]. While the management of precancerous and cancerous lesions of the bronchus have improved, early detection of such lesions is still a challenge for bronchoscopists.

Sputum cytology examination is one method to detect central type early cancer or precancerous lesions, but sometimes the origin of abnormal cells cannot be determined. Carcinoma *in situ* or early cancer are difficult to localize, because these lesions may not always show enough macroscopically recognizable changes in the bronchial mucosa [4]. Fluorescence diagnosis has been reported to improve the detection rate of intraepithelial lesions in recent years [8–10,18]. The concept of fluorescence endoscopy depends on the fact that an abnormal area has different autofluorescence from that of normal areas. The normal area of the bronchus shows green autofluorescence when excited by blue light, but abnormal areas such as cancer or a precancerous lesion show cold spots due to decreased green autofluorescence [10,11]. The LIFE system has been distributed all over the world and Lam *et al.* reported favorable results in their multicenter clinical trial [19]. In the near future, fluorescence diagnosis will be routinely used, therefore the need to make available a simple and effective device at a relatively low cost, will become necessary. One advantage of SAFE is that it uses a standard Xenon lamp with filter [14].

In the present study, the diagnostic rate between invasive cancer is same by C.B. and by SAFE. The object of fluorescence evaluation is not to diagnose advanced cancer, but the extent of the lesion could be objectively observed by SAFE, which is useful in the preoperative determination of the resection line or to determine the extent of endoscopic local treatment, such as photodynamic therapy. We encountered one out of four cases of early cancer which showed normal C.B. findings and abnormal SAFE findings. The respective sensitivity of cancer plus dysplasia and dysplasia was 66% and 51% with C.B. and 92% and 88% with SAFE. We postulated that this is a promising preliminary result for the simplified autofluorescence system. On the average, the fluorescence examination took a further 10 min after the standard bronchoscopy procedure so that this modality could be routinely used.

False positive results were frequent in cases with bronchitis both in C.B. and SAFE. C.B. tended to overdiagnose thickened bifurcation and the main reason for false negative by SAFE was due to increased cell layers by chronic inflammation, as previously reported [8,9,18].

Our study suggests the possibility of fluorescence diagnosis of subtle bronchial lesions with this simple device. Further developments of this device in a muticenter study will be necessary to prove this observation.

### *Acknowledgements*

The authors thank Professor J.P. Barron, International Medical Communications Center, Tokyo Medical University, for his excellent support in reviewing this manuscript.

### References

[1] Kato, H., Okunaka, T. and Shimatani, H. Photodynamic therapy for early stage bronchogenic carcinoma. *J. Clin. Laser Med. Surg.* 1996; **14**: 235–238.

[2] Furuse, K., Fukuoka, M., Kato, H. *et al.* A prospective study on photodynamic therapy with Photofrin II for centrally located early stage lung cancer. *J. Clin. Oncol.* 1993; **11**: 1852–1322.

[3] Hayata, Y., Kato, H., Konaka, C. *et al.* Photodynamic therapy in early stage lung cancer. *Lung Cancer* 1993; **9**: 287–294.

[4] Woolner, L.B., Fontana, R.S., Cortese, D.A. *et al.* Roentgenographically occult lung cancer-pathologic findings an frequency of multicentricity during a 10 year period. Mayo. *Clin. Proc.* 1983; **59**: 435–466.

[5] Cortese, D.A., Pairolero, P.C., Bergsralh, E.J. *et al.* Roentgenographically occult lung cancer: a 10 year experience. *J. Thorac. Cardiovasc. Surg.* 1983; **86**: 373–380.

[6] Sato, M., Saito, Y., Nagamoto, N. *et al.* Diagnositic value of differential brushing for all branches of the bronchi in patients with sputum positive or suspected positive for lung cancer. *Acta Cytol.* 1993; **37**: 879–883.

[7] Kato, H., Imaizumi, T., Aizawa, K. *et al.* Photodynamic diagnosis in respiratory tract malignancy using an excimer dye laser system. *J. Photochem. Photobiol.* 1990; **6**: 189–196.

[8] Lam, S., MacAulay, C., Hung, J., LeRiche, J. *et al.* Detection of dysplasia and carcinoma *in situ* with a lung imaging fluorescence endoscope device *J. Thorac. Cardivasc. Surg.* 1993; **105**: 1035–1040.

[9] Lam, S., Macaulay, C., Ikeda, N., LeRiche, J. *et al.* Early localization of bronchogenic carcinoma. *Diagnostic and Therapeutic Endoscopy* 1994; **1**: 75–78.

[10] Palcic, B., Lam, S., Hung, J. and MacAulay, C. Detection and localization of early lung cancer by imaging techniques. *Chest* 1991; **99**: 742–743.

[11] Hung, J., Lam, S., LeRiche, J.C. and Palcic, B. Autofluorescence of normal and malignant bronchial tissue. *Lasers Surg. Med.* 1991; **11**: 99–105.

[12] Konata, C., Auer, G., Nasiell, M. *et al.* Pathogenesis of squamous bronchial carcinoma in 20-methylchoanthrene-traeted beagle dogs. *Analyt. Quant. Cytol* 1982; **4**: 61–71.

[13] Hirano, T., Franzen, B., Kato, H. *et al.* Genesis of squamous cell lung carcinoma: sequential changes of proliferation, DNA ploidy, and p53 expression. *Am. J. Pathol.* 1994; **144**: 296-302.

[14] Kato, H., Okunaka, T., Ikeda, N. and Konaka, C. Application of simple imaging technique for fluorescence bronchoscope: prelirninary reportm ÅE *Diagnostic and Therapeutic Endoscopy* 1994; **1**: 79–81.

[15] Thiberville, L., Payne, P., Vielkinds, J. *et al.* Evidence of cumulative gene losses with progression of premalignant epithelial lesions to carcinoma of bronchus. *Cancer Res.* 1995; **55**: 133–139.

[16] Saito, M., Kato, H., Tsuchida, T. *et al.* Chemoprevention effects on bronchial squamous metaplasia by folate and vitamin B12 in heavy smokers. *Chest* 1994; **2**: 496–499.

[17] Kato, H., Okunaka, T., Konaka, C. *et al.* Photodynamic therapy for bronchogenic carcinoma. *Jpn. J. Surg.* 1997;**98**: 36–40.

[18] Ikeda, N., Kim, K., Okunaka, T. *et al.* Early localization of bronchogenic cancerous/precancerous lesions with lung imaging fluorescence endoscope. *Diagnostic and Therapeutic Endoscopy* 1997; **3**: 197–201.

[19] Lam, S., Kennedy, T., Unger, M. *et al.* Localization of bronchial intraepithelial neoplastic lesions by fluorescence bronchoscopy. *Chest* 1998; **113**: 696–702.

